# Renal Transplantation with Kidneys Affected by Tumours

**DOI:** 10.4061/2010/529080

**Published:** 2011-01-18

**Authors:** Muhammad Arslan Khurram, Aliu Oladipupo Sanni, David Rix, David Talbot

**Affiliations:** ^1^Department of Liver/Renal Transplant, Freeman Hospital Newcastle Upon Tyne NHS Foundation Trust, Newcastle Upon Tyne NE7 7DN, UK; ^2^Department of General Surgery, SUNY Downstate Medical Center, Brooklyn, NY, 11203, USA; ^3^Department of Urology, Freeman Hospital Newcastle Upon Tyne NHS Foundation Trust, Newcastle Upon Tyne NE7 7DN, UK

## Abstract

Renal transplantation confers improvement in quality of life and survival when compared to patients on dialysis. There is a universal shortage of organs, and efforts have been made to overcome this shortage by exploring new sources. One such area is the use of kidneys containing small tumours after resection of the neoplasm. This paper looks at the current evidence in the literature and reviews the feasibility of utilizing such a source.

## 1. Introduction

Renal transplantation is the optimal mode of treatment for the patients with end stage renal failure. One of the major problems for transplantation is the discrepancy between the donor and recipient numbers with far less donor than recipients. As a consequence, patients with renal failure have to wait for a long time before they can be offered an allograft. This situation is especially worse in some countries like Japan, with small cadaver programme where the average waiting time is 16 years [1].

A significant number of patients die from the complications of chronic renal insufficiency on long-term dialysis before they get a transplant. This situation is more important especially in cases where chronic kidney disease has lead to other medical problems and patient either die of the complications or become too unwell for a transplant [2]. Various measures including the use of marginal donors and use of kidneys from Maastricht category II non-heart-beating donors (NHBD) [2] have been utilized to increase the donor pool along with measures to improve and prolong graft function and survival. In addition, increasingly elderly donors are used, therefore increasing the risk of renal malignancy.

One potential area, first described by Penn [3] has been to transplant kidneys after ex vivo resection of small tumours. This was a very radical idea, because firstly, there has been evidence of transmission of donor-derived malignancy into recipient from the very early days of transplantation [4]. Secondly as a general rule, organs from donors with malignancies have not been used for the same fear with some exceptions such as central nervous system tumours [5]. Surprisingly-outcomes of the patients described in Penn's series were not as bad as could have been anticipated. 

The contemporary experience with partial nephrectomy and its success for the treatment of small renal cell cancers has lead to extrapolation of similar technique for the management of allograft malignancy [6] albeit sporadically. 

The purpose of this paper is to summarise the current evidence with regards to the utilization of kidneys with tumours for transplant and the use of conservative surgery for allografts where possible.

## 2. Material and Methods/Review Criteria

Pubmed, medline, EMBASE and CINHAL were linked searched for “renal tumour/tumor,” “kidney tumour/tumor,” “allograft tumour/tumor,” “nephron sparing surgery,” “partial nephrectomy,” and “transplant” to indentify potentially relevant articles. Articles concerning the use of kidneys after resection of renal tumour for transplant and partial nephrectomy of allograft for renal tumours were selected. References of the selected article were also searched to identify further articles of interest.

## 3. Results

From the above-mentioned criteria of the literature search the following different types of case reports/case series were identified which are discussed separately.

### 3.1. Use of Kidneys after Resection of Tumours

Normal practice when confronted with a tumour of kidney on procurement is to return it to the donor and not use any other organs [7]. In cases of deceased donors it meant that the contralateral kidney cannot be used as well because of the concerns of micro metastasis and bilaterality of some of the renal cell carcinomas (RCC).

Penn [3], reviewing the Cincinnati transplant tumour registry (CTTR), described a total of 14 cases of ex vivo resection of small renal cell cancers detected incidentally followed by transplantation. Frozen section was employed, and where margins were clear, kidneys were used although it is not clear whether all of the tumour bearing kidneys underwent frozen section. Of the cadaveric donors, the contralateral kidneys, all of which appeared healthy, were transplanted as well. Apart from these cases of renal carcinomas, there was one case of oncocytoma within the kidney which was transplanted after resection. Of all the cases where the tumour was adequately resected before transplantation there was no recurrence in a followup ranging up to 210 months. 

Buell et al. [7] presented 14 cases of transplantation after renal tumour resection from the same database as used by Penn. No recurrence has been noted up to a followup of 200 months. Median tumour size was 2.0 cm (range 0.5–4.0 cm) and all were of low histological grade. They have described two further cases since the initial data review with no recurrence and good graft function.

A similar case series from Australia [8] only included elderly recipients or those with significant co morbidities and high chance of death without transplantation. Furthermore the recipients had high levels of HLA mismatching with the donors and were selected on the basis that if there was a recurrence to occur, stopping immunosuppression may help in tumour lysis by recipient's immune response. 41 patients received kidneys after ex vivo resection of tumour of which 10 were reported as benign lesion on histopathology. One patient returned to dialysis after 30 months. 4 patients died of unrelated causes. There was only one recurrence noted 9 years after transplantation out of the remaining 30 patients. Notably this tumour recurrence was at a distance from the initial resection site, this therefore may not be a tumour recurrence but another primary within a “field” change renal tissue. The patient refused any further treatment, and the lesion has grown 0.2 cm in 18 months since diagnosis. In a followup study on these patients this group has recently published long-term outcomes which are significantly better than wait-listed patients on dialysis and are comparable to the live unrelated transplants [9].

Mannami et al. [10] from Japan published a series of 42 “restored” kidneys from live donors. Eight donors with small renal cell carcinoma (<3.5 cm) underwent donor nephrectomy and ex vivo resection of the tumour followed by transplantation of the kidney. Five patients were alive, three with functioning grafts, two died with functioning grafts from unrelated caused, and one was lost to followup. No tumour recurrence has been noted in any of these patients. Another 8 patients had donor nephrectomies which had benign diseases of which 5 had partial resection and kidney used for transplantation. Three recipients are alive with functioning grafts, while four have gone back to dialysis (after 3,18,51,73 months). One recipient died of unrelated pathology.

There have also been 6 case reports [11–16] of live related kidney donation when a tumour was detected incidentally in or ex vivo and the kidney was transplanted after resection. No recurrence has been noted in any of these cases with a followup of up to more than 10 years.

### 3.2. Partial Nephrectomy for Tumours Diagnosed after Transplantation

Renal cell carcinoma represents around 4.6% of all the tumours in allograft recipients with only 10% of these occurring in the allograft itself [3].

The other main subgroup is when a tumour was detected after transplant. Again the standard practice here has been to perform transplant nephrectomy [17] with the patient invariably returning to dialysis and normally being put on a waiting list for another transplant if feasible. 

Until now, more than 50 cases of allograft renal cell tumours have been described in the literature of which at least 35 cases have had nephron sparing surgery (NSS) for their allograft tumour [6, 11, 18–36]. Tumour sizes have range, from 0.5 to 4.0 cm although there have been two case reports of larger (6–8 cm) tumours all being successfully treated with NSS [18, 19]. Postoperative followup is from one month to more than 10 years with one recurrence 5 years after NSS in renal allograft [37]. This was in a 74 year old recipient five years after initial transplant. A 2.4 cm RCC was incidentally detected without any evidence of distant metastasis. It was treated with radical nephrectomy and patient has been disease free on hemodialysis.

### 3.3. Contralateral Transplanted Kidney with a Renal Tumour in Cadaveric Donor

These kidneys again are normally not used as RCC can be bilateral especially the papillary subtype [38]. Penn [3] has described 14 cases in which the contralateral kidney was transplanted from patients with renal tumour. One patient had recurrence in the allograft which was removed for rejection. This patient died 75 months after transplantation from a de novo cancer of one of his own kidneys. The remaining patients did not have any recurrence with a followup ranging from 0.5 to 153 months.

Nicol et al. [8] described 2 similar cases with no recurrence. Barrou et al. [38] has described a case of two allograft recipients from a single donor with tubulopapillary tumour (17 mm) in the right kidney; only the left kidney was utilized for transplantation. Shortly after transplantation, the recipient underwent an ultrasound (US) examination of the allograft which did not reveal any tumour. 3 months later a biopsy was done for rejection which revealed a poorly differentiated tumour and the patient underwent radical allograft nephrectomy. No additional chemotherapy was given apart from discontinuation of immunosuppression (prednisolone and azathioprine). Lymph nodes that had been noted to be enlarged on CT scan disappeared two month after nephrectomy. The patient underwent re transplantation two years later and was disease free and dialysis independent at 3 year followup. Another patient received the heart transplant from the same donor but died from bony metastasis from the renal cell carcinoma.

### 3.4. Accidental Transplantation

In at least 4 cases [3, 13, 20, 39] there have been accidental transplantation of RCC mistaken as a benign pathology on procurement. Partial nephrectomy/enucleation in all these cases was performed before transplantation with adequate resection margins. Routine histopathology revealed the resected tumour to be malignant. All recipients retained the allograft because of complete excision of the tumour and were kept under close follow-up with no recurrence so far.

The cases where there have been transplantation of tumour, either partially resected or unrecognized at the time of transplant have resulted in disastrous outcomes [3, 38].

### 3.5. Miscellaneous

Manammi et al. [10] reported a series of 8 patients who underwent nephrectomy for a distal ureteric transitional cell carcinoma (TCC). One patient had a recurrence of TCC after 15 months and was offered graft nephrectomy but opted for partial resection of ureteric tumour to prevent returning to dialysis. He died three years after partial resection from a squamous cell carcinoma of lung with liver metastasis. His TCC also had squamous metaplasia and a DNA study to determine exact origin of primary tumour could not be established because of inadequate tissue samples. The remaining patients were either alive with functioning grafts or died of unrelated causes.

### 3.6. Opinion of Patients and Transplant Specialists

Transplantation of kidneys with cancers is a novel idea not only among patients but also among the transplant community. To be able to exploit this potential donor pool it is of utmost importance that both the health care specalists; transplants surgeons and nephrologists and the patients both donors and recipients are comfortable with the idea of using such kidneys. To determine this, structured questionnaires were sent to focus group of patients on the North East renal transplant waiting list, postnephrectomy patients for small renal cancer, nephrologists and transplant surgeons in the UK [40]. 

Results are shown in Table 1 and have a generally high response rate. Those respondents that had lost their kidney, removed for tumour, had the highest consent rate and patients potentially receiving such kidney the lowest. The transplant surgeon and nephrologists had views somewhere in between. 

This survey was done in UK from where there have been no case reports of using organs after removal of tumour and but still the response was largely favourable. Given that since this survey there has been an increase in total number of such organs being utilized, one can extrapolate that current belief may be more favourable.

### 3.7. Role of Immunosuppression

One of the worries about transplantation of tumour affected kidneys is the potential of tumour recurrence and growth in state of potential immune inattention due the immunosuppressive therapy. Renal cell carcinoma is known to be an immunogenic tumour [21] but in the presence of immunosuppression, if there was any transplantation of tumour cells in the host, then the potential of continued growth will be higher in a host with a compromised immune system. Furthermore, immunosuppression in itself has been known to increase the incidence of de novo malignancy [41, 42]. Because of these concerns, an immunosuppressive agent with no potential to increase de novo malignancy and better still to have antitumour activities would probably be ideal. Rapamycin has shown some promise as being a protective agent against RCC progression [21, 43, 44].

## 4. Discussion

Incidence of RCC has increased in Western countries in the last few years owing to the widespread use of US and CT scanning [45, 46]. Most RCC are now picked up at an early stage on investigations done for other reasons [47]. Furthermore the incidence of RCC in allografts will continue to increase as older people donate organs and graft survival is improved by better immunosuppression. Longitudinal studies have shown that many small tumours have a slow growth pattern with low metastatic spread in tumours of <3 cm [48, 49]. Autopsy studies have shown that RCC are present in 1%–20% of patients dying from unrelated cases, meaning that many of the tumours will not prove to be clinically significant in the course of patient's life [50, 51].

The gold standard treatment of resectable renal cell carcinoma has been radical nephrectomy. Recent evidence has changed this practice dramatically as survival after radical nephrectomy (RN) and partial nephrectomy (PN) has shown to be comparable [52]. Favourable outcomes have been observed after NSS for <4 cm RCCs and RN has been described as “surgical overkill” [53] for these tumours. Furthermore, local recurrence after NSS has been reported to be <5% with recurrences mostly associated with large and multifocal tumours. 

A significant risk of dying in patients on dialysis particularly in older patient has been one of the driving forces to increase the number of kidney donors. Renal transplantation seems to confer a substantial survival advantage over dialysis in patients with end-stage renal failure [2]. A significant number of patient accepted for dialysis are older patients, who have a mortality risk of 25%. With longer waiting times for a transplant, it is inevitable that many of the patients will die before they can receive a transplant which would have improved their quality of life and longevity [2]. Furthermore 16% to 23% of suspicious lesion resected from kidneys are either benign or of low malignant potential [53–55] and not using these kidneys with small tumours after partial nephrectomy for transplantation seems wastage of precious organs when one considers the benefits of transplantation over dialysis.

 A suspicious lesion found at multiorgan retrieval should have an excision biopsy and histological confirmation of clear margins before any of the organs can be transplanted. A malignant lesion in the kidney when unrecognized and transplanted continues to grow under the immunosuppression carries high risk of metastasis and can result in fatal outcome. If the biopsy confirms clear margins with favourable histology then these organs could be used for transplantation as risk of recurrence is very low. Situation is more complex when it comes to using restored organs from live (related/unrelated) renal cell carcinoma patients. Major difference being that these are live cancer patients first and therefore must never be treated primarily as potential organ donors to prevent any bias in treating their primary problem which may lead to provision of less than optimal treatment and ultimately harm to these patients [8, ed]. This is shown by Takahara et al. [56] in their review of Mannami et al. series concerning ureteric carcinoma patient, where adherence to standard practice for treating these tumours was not practiced with disastrous consequences. With changing trends, radical nephrectomy is now regarded as an alternate standard of care to partial nephrectomy for T1a tumours when partial nephrectomy is not technically feasible. This is due to the comparable oncological outcomes after partial nephrectomy and evidence that radical nephrectomy is an independent predictor of low GFR. A positive outcome for a recipient can never justify harm to a live donor; on the contrary, for a transplant with a live donor to be regarded as a success means that both the recipient and the donor have done well [57]. Live related donors in Nicol et al. series were given the options of observation, radical or partial nephrectomy without any mention of the possibility of use of organs for transplantation. Only after the patients had decided to opt for radical nephrectomy possibility of domino donation was discussed. This approach has the benefit of making sure that patients make their own decisions without any pressure from clinicians. Other important factor is to make sure that beliefs of the clinician do not affect patient's treatment choices. Importance of detailed informed consenting cannot be over emphasised for the recipients of such restored organs. All the relevant information especially of the origin of the organ and potential of recurrence and associated risk must be discussed fully and patients understanding checked. 

Routine followup of the patients with annual US have been suggested to make sure any recurrence is diagnosed as early as possible. Tumours have been detected at early stage with better outcomes because of regular followups. If one kidney is found to have a tumour it is important that the other kidney is closely followed up. It is easier in the live donor setting when the donor can be carefully followed up but in cadaveric donation there has to be a central database for tracking the contralateral kidney [22] which might be transplanted into a recipient in a different unit. 

Immunosuppression is essential after transplant and unfortunately this has been associated with the higher incidence of cancers in recipients as opposed to the general population [42]. Certain newer immunosuppressive agents have anti tumour [58] activity and their use can, in theory not only reduce the chances of recurrence but they can also be used to treat patient should a recurrence occur. 

Furthermore Human Tissue Act 2004 [59] that covers the use of organs for transplant in the UK allows anyone to be a donor including live related and unrelated (altruistic donor) provided there is adequate consenting. This means that donation can also occur from patients suffering from small renal cell carcinoma who have radical nephrectomy as primary treatment provided measures are taken to ensure that these patients are treated appropriately in the first place and both donor and recipients had given informed consent.

## 5. Conclusion

To increase the donor pool new sources have to be exploited. Use of kidneys after tumour resection seems a feasible source. There are several important issues in using such marginal and potentially dangerous organs; patients should have complete understanding of the implications of the type of organ they are donating and receiving, good surgical technique and rigorous pathological testing of the resected tissue to make sure there is no tumour left behind, regular followup with adequate investigations, and a reliable organ tracking system to investigate the recipient of contralateral organ should one organ develop a recurrence. On top of this, transplant surgeons and nephrologists should be comfortable in using such organs. Usage of such organs is still in its infancy, and for a much wider acceptance of this source to occur, there is need for more research. One interesting area will be to explore the new immunosuppressive agents with antiproliferative properties on such recipients with the potential to reduce recurrence rate or better still to prevent it altogether while either replacing standard immunosuppressive agents or reducing their required dose thereby reducing side effects. 

## Figures and Tables

**Table 1:** 

Respondents	Response rate	Support use of kidneys
Potential recipients on waiting list	97% (113/116)	59% (67/113)
Previous nephrectomy (potential donors)	100% (15/15)	93% (14/15)
Nephrologists	58% (94/161)	78% (73/94)
Transplant surgeons	66% (43/65)	72% (31/43)
